# Pan-Cancer Computational Analysis of RKIP (*PEBP1*) and LKB1 (*STK11*) Co-Expression Highlights Distinct Immunometabolic Dynamics and Therapeutic Responses Within the Tumor Microenvironment

**DOI:** 10.3390/ijms26157145

**Published:** 2025-07-24

**Authors:** Evangelia Skouradaki, Apostolos Zaravinos, Maria Panagopoulou, Ekaterini Chatzaki, Nikolas Dovrolis, Stavroula Baritaki

**Affiliations:** 1 Laboratory of Experimental Oncology, Division of Surgery, School of Medicine, University of Crete, 71003 Heraklion, Greece; evaskouradaki@gmail.com; 2 Cancer Genetics, Genomic and Systems Biology Group, Basic and Translational Cancer Research Center (BTCRC), 1516 Nicosia, Cyprus; a.zaravinos@euc.ac.cy; 3 Department of Life Sciences, School of Sciences, European University Cyprus, 2404 Nicosia, Cyprus; 4 Laboratory of Pharmacology, Department of Medicine, Democritus University of Thrace, 68100 Alexandroupolis, Greece; mpanagop@med.duth.gr (M.P.); achatzak@med.duth.gr (E.C.); 5 Institute of Agri-Food and Life Sciences, Hellenic Mediterranean University Research Centre, 71410 Heraklion, Greece

**Keywords:** RKIP, LKB1, cancer, tumor microenvironment

## Abstract

RKIP and LKB1, encoded by *PEBP1* and *STK11*, respectively, have emerged as key regulators of cancer pathophysiology. However, their role in shaping tumor progression through modulation of the tumor microenvironment (TME) is not yet fully understood. To address this, we performed a comprehensive pan-cancer analysis using TCGA transcriptomic data across 33 cancer types, grouped by their tissue of origin. We investigated *PEBP1/STK11* co-expression and its association with transcriptomic reprogramming in major TME components, including immune, mechanical, metabolic, and hypoxic subtypes. Our results revealed both positive and inverse correlations between *PEBP1/STK11* co-expression and TME-related molecular signatures, which did not align with classical cancer categorizations. In a subset of tumors, *PEBP1/STK11* co-expression was significantly associated with improved overall survival and reduced mortality (HR < 1). Notably, we predominantly observed inverse correlations with pro-inflammatory and immunosuppressive chemokines, immune checkpoints, extracellular matrix components, and key regulators of epithelial-to-mesenchymal transition. In contrast, we found positive associations with anti-inflammatory chemokines and their receptors. Importantly, *PEBP1/STK11* co-expression was consistently linked to reduced expression of drug resistance genes and greater chemosensitivity across multiple tumor types. Our findings underscore the co-expression of *PEBP1* and *STK11* as a promising target for future studies aimed at elucidating its potential as a biomarker for prognosis and therapeutic response in precision oncology.

## 1. Introduction

The tumor microenvironment (TME) represents a highly complex and dynamically evolving biological system, with distinct compositional differences across various tumor types [1]. It primarily consists of cellular elements and non-cellular/structural components including immune and stromal cells, blood vessels, and the extracellular matrix (ECM) [2,3]. Recently, the TME landscape has been recognized as encompassing distinct specialized niches that interact and overlap, engaging in dynamic crosstalk [4]. These specialized microenvironments can be systematically classified into six major types: hypoxic niche, immune microenvironment, metabolic microenvironment, acidic niche, innervated niche, and mechanical microenvironment [4,5,6]. The intricate interplay among the various TME sub-microenvironments not only regulates and sustains tumor survival and growth, but also facilitates the acquisition of an invasive phenotype by cancer cells. This, in turn, enables their dissemination from the primary site and migration to distant locations through a complex, multistep metastatic cascade [3,7]. Furthermore, the TME significantly influences cancer responses to conventional chemotherapeutics by modulating drug sensitivity, resistance mechanisms, and immune evasion, ultimately impacting treatment efficacy [8].

Over the years, numerous gene products have been identified as onco- and metastasis-inducers, with their expression in cancer, immune, and stromal cells being strongly influenced by TME crosstalk. However, only a few have been recognized as endogenous onco-suppressors that specifically function to suppress metastasis and/or drug resistance. Among them the Raf kinase inhibitory protein (RKIP), encoded by Phosphatidylethanolamine Binding Protein 1 (*PEBP1*), has been identified by us and others as a key regulator of intracellular signaling pathways involved in physiological processes and disease pathogeneses, such as cancer [9,10]. In various cancer types RKIP functions as a direct suppressor of key signaling pathways including Raf-1/Mek/Erk, NF-κB, STAT3, GSK3β, and GPCRs signaling. This suppression leads to a reduction in cancer cell proliferation and survival, as well as attenuation of various stages of the multifaceted metastatic cascade, including epithelial-to-mesenchymal transition (EMT) [11,12]. One of the most notable cancers where RKIP acts as a metastasis suppressor is breast cancer, where it inhibits angiogenesis, local invasion, intravasation, and colonization [13,14,15].

RKIP further appears to counteract tumor resistance to chemotherapy, immunotherapy, and radiotherapy, while enhancing anticancer host immunosurveillance [11] via direct suppression of the aforementioned signaling pathways [11,12,16,17]. In this context, tumors exhibiting reduced RKIP expression, are often associated with increased resistance to conventional chemotherapeutics, thus suggesting that RKIP could serve as a potential predictive biomarker for chemotherapy responsiveness, while therapeutic strategies aimed at upregulating RKIP expression may enhance treatment efficacy and improve clinical outcomes [18]. Accordingly, RKIP levels have been positively associated with improved clinical outcomes in multiple cancers such as breast, prostate, melanoma, glioma, colorectal, lung, pancreatic, hepatocellular carcinoma, gastric, ovarian, liver, kidney, and other solid and hematological malignancies [11,14,15,19,20,21,22,23,24,25].

The diminished or even complete loss of RKIP expression has been observed in a wide range of solid and hematological malignancies [11] while this reduction has been strongly associated with significantly shorter overall patient survival [26]. Moreover, studies have established a causal relationship between RKIP levels and several proteins involved in TME regulation, thus suggesting that RKIP reduction may contribute to the remodeling of the TME in a way that supports tumor progression [27]. Multiple regulatory mechanisms have been described in the literature in contributing to RKIP loss, including promoter hypermethylation via EZH2-mediated histone modifications, transcriptional repression by BACH1 and Snail1 [11,17,28], post-transcriptional regulation via microRNAs, like miR-224, and long non-coding RNAs [29,30,31], as well as post-translational modifications, such as phosphorylation at serine 153 by protein kinase C, which leads to the loss of RKIP activity [11,32,33]. Given its critical role in tumor biology, RKIP levels could therefore be clinically significant, serving both as a prognostic marker for tumor aggressiveness and a potential therapeutic target. In this context, strategies aimed at restoring RKIP expression levels and activity hold promise for improving cancer treatment outcomes.

Liver Kinase B1 (LKB1), encoded by the Serine–Threonine Kinase 11 (*STK11*) gene, is another example of a tumor suppressor whose expression and activity are frequently subject to loss or inactivation in various malignancies, thus contributing to tumorigenesis and disease progression [34,35]. LKB1 activation is controlled by an allosteric mechanism, where it forms a heterotrimeric complex with two accessory proteins, the STE20-related kinase adaptor (STRAD), and mouse protein 25 (MO25), also referred to as calcium binding protein 39 (CAB39) [36,37]. This protein functions primarily as a key kinase that phosphorylates and activates AMP-activated protein kinase (AMPK), a central energy sensor that responds to changes in nutrient availability and cellular energy stress [34,38]. As such, LKB1 plays a vital role in regulating essential cellular processes, including cell migration, proliferation, and metabolism [39]. Furthermore, both LKB1 and AMPK are involved in maintaining cell polarity, whose disruption is implicated in carcinogenesis [40,41,42].

Germline mutations in *STK11* are predominantly linked to a hereditary, autosomal dominant cancer predisposition syndrome characterized by gastrointestinal (GI) polyposis, distinctive mucocutaneous pigmentation, and an elevated risk of malignancy [43,44]. Individuals diagnosed with Peutz–Jeghers syndrome (PJS) exhibit a significantly increased susceptibility to developing GI neoplasms, including colorectal, pancreatic, and gastric cancers, along with other non-GI epithelial malignancies, such as breast, uterine, and cervical cancers, lung cancer, and gonadal tumors affecting the ovaries and testes [45,46].

LKB1 is one of the few serine/threonine kinases frequently inactivated by mutations during cancer development [40]. Loss of LKB1 function enables tumors to evade cancer immunosurveillance through several mechanisms [47]. One such mechanism involves the disruption of LKB1′s role in preventing the epigenetic silencing of STING, a crucial component of the innate immune response. Normally, LKB1 inhibits DNA methyltransferase activity, thereby maintaining STING expression. In LKB1-deficient tumors, increased DNA methylation leads to STING repression, impairing downstream anti-tumor immune signaling. This inactivation of STING represents a novel immune evasion strategy, particularly in lung adenocarcinomas harboring co-mutations in KRAS and *STK11*, where it contributes to resistance against host immune responses [48,49,50]. Additionally, LKB1 loss has been shown to induce complex alterations in the TME, indicating its involvement in angiogenesis regulation and potential effects on tumor response to anti-angiogenic therapy [47,51]. Studies also suggest that LKB1 promotes resistance to gemcitabine in the MDA-MB-231 breast cancer cell line, potentially by facilitating gemcitabine breakdown and shielding cells from DNA damage [52]. Furthermore, other preclinical studies have shown that LKB1 loss reduces the sensitivity of tumor cells to radiotherapy [53,54].

Along with RKIP, LKB1 is also considered a key metastatic suppressor in various cancer types, including lung, breast, ovarian, colorectal, and prostate cancer [55,56,57,58,59,60]. Studies have revealed that low LKB1 expression promotes tumor metastasis by regulating EMT through the p38 MAPK signaling [61]. In this context, a distinct mechanism for LKB1-mediated adhesion involves the LKB1-NUAK1 pathway, which regulates cell detachment and adhesion through myosin light chain 2 (MLC2) and myosin phosphatase (MYPT1). Inhibition of this pathway disrupts cell detachment and enhances adhesion [62]. Additionally, expression profiling in human lung cancer cell lines and mouse lung tumors revealed several metastasis-promoting genes, including *NEDD9*, *VEGFC*, and *CD24*, as targets of LKB1 repression [63].

At a clinical level, previous studies have shown that LKB1 expression is associated with improved survival in patients with advanced non-small cell lung cancer receiving chemotherapy and bevacizumab treatment [64], while the loss of tumoral LKB1 expression serves as a negative prognostic indicator in patients with operable colorectal cancer [65]. Furthermore, LKB1 expression has been identified as a potential predictor of overall survival in a subset of HER2-positive patients [66].

The well-established tumor-suppressive functions of RKIP (*PEBP1*) and LKB1 (*STK11*) suggest that their expression, either independently or in concert, may influence cancer progression through effects on the tumor microenvironment (TME). Given the TME’s structural and functional complexity—comprising immune, metabolic, hypoxic, and mechanical components—understanding how these genes might interact with distinct microenvironmental niches could inform future studies on tumor behavior and treatment response. While both genes have been individually implicated in TME modulation, to our knowledge, their combined expression patterns across cancer types and potential associations with specific TME features have not been systematically characterized.

In this study, we use a computational, transcriptomics-based pan-cancer approach to explore whether co-expression of *PEBP1* and *STK11* is associated with distinct TME profiles. Rather than asserting causality, our goal is to integrate prior biological knowledge with public omics data to generate testable hypotheses that can guide future mechanistic and translational research.

## 2. Results

Our study employed an integrative bioinformatic and computational framework to systematically investigate the co-expression patterns of *PEBP1* (RKIP) and *STK11* (LKB1) transcripts across 33 distinct malignancies retrieved from TCGA datasets (accessed on 17 May 2025). This analysis was conducted in the context of cancer-type–specific pathophysiological characteristics and TME features and functions. Through this approach, we identified several significant positive and negative associations between RKIP/LKB1 co-expression and key clinicopathological parameters, including chemoresistance and metastatic potential, as well as with cellular and non-cellular components of the TME spanning various niches. Notably, many of these associations exhibited clear cancer-type–specific patterns. These integrative findings provide a computationally driven basis for formulating hypotheses that could be experimentally validated to explore their putative clinical relevance.

### 2.1. PEBPI/STK11 Co-Expression Favors Overall Survival in a Cancer-Type Dependent Manner

**Figure 1:**
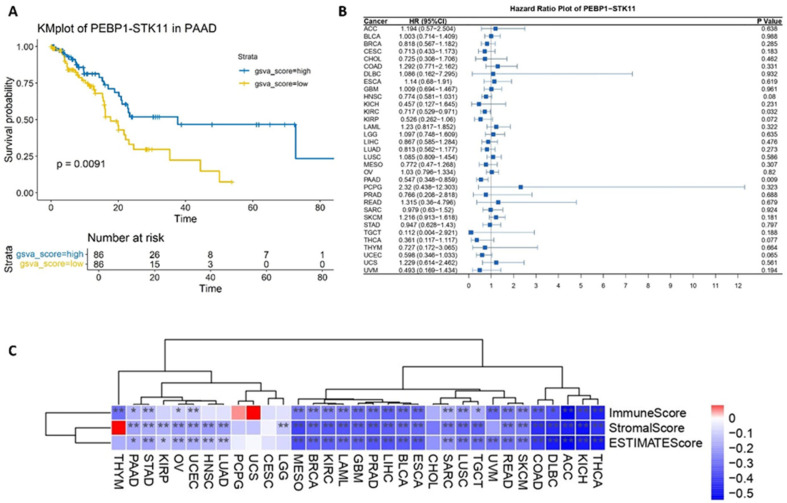
Impact of *PEBPI/STK11* co-expression on clinicopathological characteristics at the pan-cancer level. (**A**) Overall survival analysis in pancreatic adenocarcinoma (PAAD), (**B**) Hazard Ratio analysis across multiple cancer types, and (**C**) ESTIMATE score correlation analysis. Color coding: red denotes a positive correlation and blue denotes an inverse (negative) correlation. * *p* ≤ 0.05, ** *p* ≤ 0.01.

Overall survival (OS) and Hazard Ratio (HR) analyses were conducted within a pan-cancer framework to assess the impact and the putative clinical significance of the co-expression of the *PEBP1* and *STK11* transcripts. Notably, our findings revealed a significant positive correlation of *PEBP1/STK11* co-expression and OS in pancreatic ductal adenocarcinoma (PAAD) exclusively, suggesting a potentially distinct role of these genes in PAAD progression and survival probability (Figure 1A). Similarly, the HR analysis identified statistically significant associations in both PAAD and clear cell renal carcinoma (KIRC), with HR values of 0.547 and 0.717, respectively. These findings imply a potential protective role for *PEBP1* and *STK11* co-expression in the progression of these cancers, potentially reducing the mortality risk (Figure 1B).

To further investigate the role of *PEBP1* and *STK11* co-expression within the tumor microenvironment (TME), we conducted a comprehensive set of analyses. First, we performed an ESTIMATE score analysis, which quantifies the relative abundance of non-tumor cellular components, including immune and stromal cells, within the tumor. The results showed a statistically significant negative correlation between the ESTIMATE score and *PEBP1* and *STK11* co-expression patterns across the majority of cancer types. Notably, this correlation was absent in cholangiocarcinoma (CHOL), cervical carcinoma (CESC), uterine carcinosarcoma (UCS), glioblastoma multiforme (GBM), paraganglioma and pheochromocytoma (PCPG), and thymoma (THYM). Additionally, the stromal score, which reflects the proportion of stromal components within the tumor, also exhibited a negative correlation with the co-expression of the aforementioned genes. However, no such correlation was detected in CHOL, CESC, UCS, PCPG, THYM, and uveal melanoma (UVM) (Figure 1C).

### 2.2. LKB1 Exhibits Broader and Stronger Functional Associations with TME Subtypes Compared to RKIP Based on a Multiple-UniReD Analysis

To start with, we explored the potential roles of RKIP and LKB1 protein expression in distinct functional aspects of the TME by utilizing the literature mining tool Multiple UniReD to assess functional associations, as previously described in [67]. A curated reference list of proteins known to influence drug resistance, hypoxia, metabolism, and the mechanical properties of the TME, was used to represent characteristic sub-microenvironments (niches), namely, hypoxic, metabolic, and mechanical (Supplementary Figure S1). LKB1 demonstrated strong associations across multiple sub-microenvironments, with a score of 13.5 out of 15 (90%) for drug resistance, 39 out of 50 (78%) for the mechanical TME, 28 out of 43 (65.1%) for hypoxia, and a moderate score of 20.5 out of 50 (41%) for the metabolic TME. In comparison, RKIP showed its strongest association with the mechanical TME (33 out of 50, 66%), followed by drug resistance (9.5 out of 15, 63.3%), hypoxia (26 out of 43, 60.5%), and a notably weaker association with the metabolic TME (9.5 out of 50, 19%).

Overall, the UniReD analysis revealed more extensive and robust associations for LKB1 than for RKIP, particularly in relation to drug resistance and mechanical properties of the TME.

### 2.3. PEBP1/STK11 Co-Expression Associates with the Molecular Remodeling of Distinct TME Niches

We further investigated in detail the impact of *PEBP1* and *STK11* co-expression on the molecular remodeling of distinct TME niches, including the immune, metabolic, hypoxic, and mechanical sub-microenvironments, in the context of their cellular and non-cellular components.

#### 2.3.1. Immune Niche

##### Immune Cell Infiltration: Immune Score

**Figure 2:**
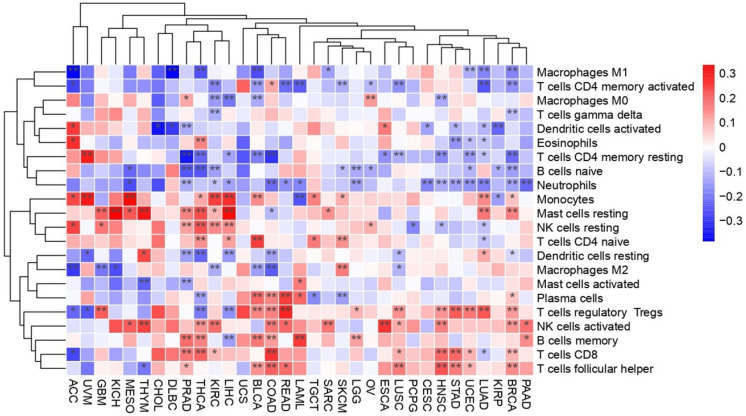
Heatmap illustrating the transcriptional impact of *PEBP1/STK11* co-expression on alternations in immune cell infiltration across cancer types. Color coding: red, positive correlation; blue, inverse (negative) correlation. * *p* ≤ 0.05, ** *p* ≤ 0.01.

Innate immune cells, including macrophages, dendritic cells, eosinophils, natural killer cells, neutrophils, monocytes, and mast cells, as well as, adaptive immune cells like CD8+ T cells, CD4+ naïve and memory T cells, regulatory T cells, gamma–delta T cells, helper T cells, plasma cells, and naïve and memory B cells, were analyzed to assess immune cell infiltration patterns in relation to the co-expression of *PEBP1/STK11* (Figure 2). Among the immune cell populations of the innate immunity, macrophages of the M2 phenotype showed a negative correlation with the co-expression of *PEBP1/STK11* in several cancer types, including colorectal adenocarcinoma (COAD), bladder urothelial carcinoma (BLCA), chromophobe renal cell carcinoma (KICH), KIRC, GBM, and lung squamous cell carcinoma (LUSC). In contrast, a strong positive correlation was observed in skin cutaneous melanoma (SKCM). No statistically significant correlation was identified in the other cancer types analyzed. Natural killer (NK) cells, both in their resting and activated states, displayed a positive correlation with the co-expression of *PEBP1/STK11* across a broad spectrum of cancers, predominantly within the gastrointestinal tract (excluding CHOL and gastric adenocarcinoma (STAD)). Additionally, positive correlations were observed in KIRC, breast ductal carcinoma (BRCA), ovarian serous cystadenocarcinoma (OV), prostate adenocarcinoma (PRAD), LUSC, mesothelioma (MESO), head and neck squamous cell carcinoma (HNSC), thyroid papillary carcinoma (THCA), THYM, and sarcoma (SARC). Neutrophils on the other hand exhibited a negative association with *PEBP1/STK11* co-expression in most cancer types, particularly in gastrointestinal cancers (excluding CHOL and esophageal carcinoma (ESCA)), BRCA, CESC, PRAD, uterine corpus endometrioid carcinoma (UCEC), KIRC, lower grade glioma (LGG), lung adenocarcinoma (LUAD), MESO, HNSC, and acute myeloid leukemia (LAML).

Regarding the infiltration of the adaptive immune cell populations, the presence of CD8^+^ T cells within the TME was positively correlated with the *PEBP1/STK11* co-expression patterns in COAD, STAD, KIRC, BRCA, UCEC, LUSC, HNSC, and THCA. However, negative correlations were identified in PRAD and LUAD, suggesting that the impact of these genes on cytotoxic T-cell infiltration may vary depending on the tumor type. Similarly, T helper cells exhibited a positive correlation in COAD, rectum adenocarcinoma (READ), BLCA, BRCA, UCEC, LUSC, and HNSC, while a negative correlation was observed in PRAD and THYM. indicating potential immunomodulatory differences across these malignancies. Furthermore, our analysis revealed a complex, state-dependent intra-relationship between B cell infiltration and *PEBP1/STK11* co-expression. This dynamic interaction appears to vary based on the activation or differentiation status of B cells, with distinct profiles emerging for naive, memory, or plasma cell subtypes. It suggests that RKIP and LKB1 may modulate B cell functions differently, potentially influencing immune responses within the TME. A similar dynamic relationship was also seen in CD4+ T cells, where the correlation nature also depended on the activation or differentiation state of CD4+ T cells, with distinct patterns emerging for naive, activated, memory, or resting memory subtypes.

Lastly, resting mast cells demonstrated a strong positive correlation with *PEBP1/STK11* co-expression of in multiple cancers, including hepatocellular carcinoma (LIHC), KICH, KIRC, BRCA, PRAD, LUAD, MESO, THCA, and SARC. However, a significant negative correlation was found exclusively in COAD and GBM.

Accordingly, the immune score analysis which estimates the level of immune cell infiltration within a tumor, exhibited a negative trend across most cancers. However, no significant correlation was observed in CHOL, papillary renal cell carcinoma (KIRP), CESC, UCS, LGG, LUAD, HNSC, and PCPG cancers (Figure 1C).

##### Inhibitors of Immune Responses

Immune checkpoints are integral non-cellular components within TMEs that govern immune cell function via maintaining self-tolerance through activating inhibitory pathways [68]. Given their significance in immune homeostasis, understanding how *PEBP1/STK11* co-expression influences immune checkpoint regulation is of considerable importance. Our analysis revealed a widespread negative correlation between the co-expression of these genes and the expression of genes encoding immune checkpoint molecules, such as *CD274*, *PDCD1LG2*, *PDCD1*, *CTLA4*, *TIGIT*, and *CD244*, across multiple cancer types, including gastrointestinal, reproductive, nervous, respiratory, ocular, and hematopoietic cancers. Among other genes that encode immune inhibitors, *IL10*, *TGFBR1*, and *IDO1* exhibited a broadly negative correlation across most cancer types, aligning with the expression pattern of *PEBP1/STK11*. This finding suggests that the co-expression pattern of *PEBP1/STK11* may influence immune evasion mechanisms within tumors. However, a few exceptions were noted, highlighting the potential for context-specific regulatory effects.

**Figure 3:**
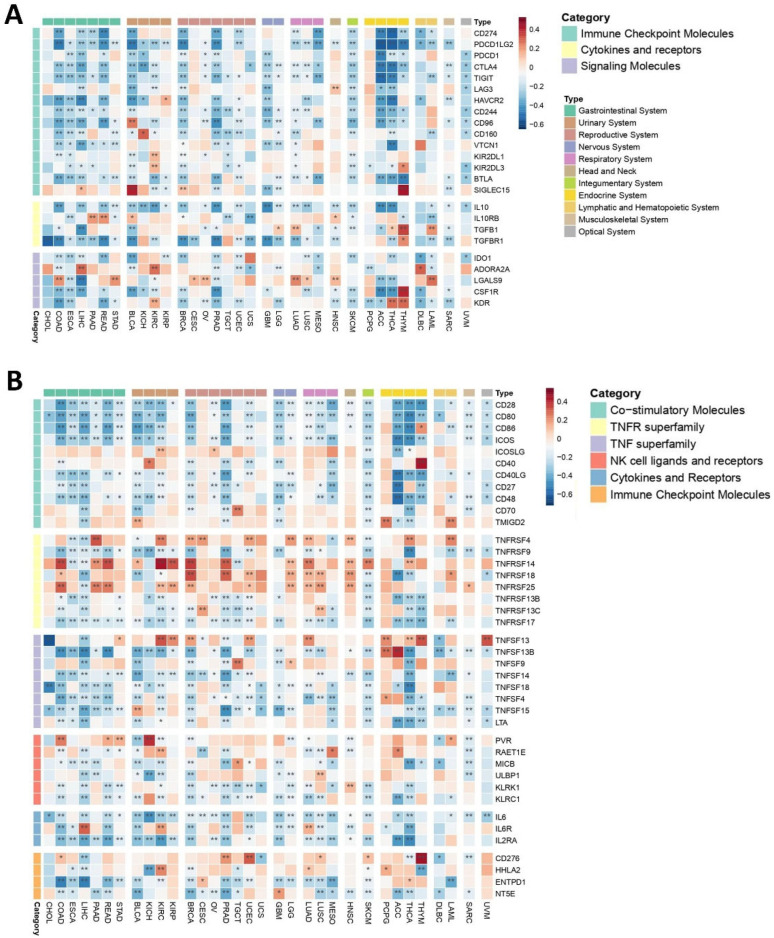
Heatmaps depicting the associations between *PEBP1/STK11* co-expression and the transcriptional levels of immune modulators. (**A**) Immune inhibitory genes and (**B**) immune stimulatory genes. Color coding: red, positive correlation; blue, negative correlation. * *p* ≤ 0.05, ** *p* ≤ 0.01.

In contrast, genes encoding immune inhibitors such as *IL10RB*, *ADORA2A*, *LGALS9*, *CSF1R*, and *KDR* demonstrate a more intricate and variable correlation pattern. Depending on the cancer type, these inhibitors genes can show either positive or negative correlations, suggesting a context-dependent regulatory phenotype (Figure 3A).

##### Immune Stimulators

Most of the immune stimulators analyzed demonstrated a complex, cancer type-dependent expression pattern, suggesting that their correlation with *PEBP1/STK11* is influenced by the specific TME of each cancer (Figure 3B). Notably, most of Tumor Necrosis Factor (TNF) receptor genes including *TNFRSF4*, *TNFRSF14*, *TNFRSF18*, *TNFRSF25*, as well as *CD276*, *HHLA2*, *PVR*, and *RAET1E*, were among the genes encoding immune stimulators displaying the strongest positive correlations with *PEBP1/STK11* co-expression. In contrast, a uniform negative association with *PEBP1/STK11* co-expression was found in most of the genes encoding TNF ligands (*ENFSF13B*, *TNFSF4*, *TNFSF9*, *TNFSF14*, *TNFSF18*, and *TNFSF15*) in almost all cancer types. In general, at least one of the genes that encode immune stimulators exhibited a positive correlation with *PEBP1/STK11* expression across all cancer types, with the exception of CHOL, ESCA, UCS, and diffuse large B-cell lymphoma (DLBC). The cancer type showing the most positive correlations, particularly with *ICOSLG*, *CD40*, *TNFRSF4*, *TNFRSF14*, *TNFRSF25*, *TNFSF13*, *HHLA2*, and *RAET1E*, was KIRC. Additional malignancies, including COAD, UCEC, LGG, HNSC, PCPG, and respiratory cancers, also exhibited numerous positive correlations. In contrast, other genes that encode immune stimulators, such as *CD28*, *CD80*, *ICOS*, *CD40LG*, *CD27*, *CD48*, *IL6*, *IL2RA*, *LTA*, *MICB*, and *KLRC1* generally showed a negative correlation with *PEBP1/STK11* co-expression across most cancer types.

These findings underscore the intricate and cancer-specific interactions between immune stimulators and *PEBP1/STK11* expression.

##### Chemokines and Chemokine Receptors

**Figure 4:**
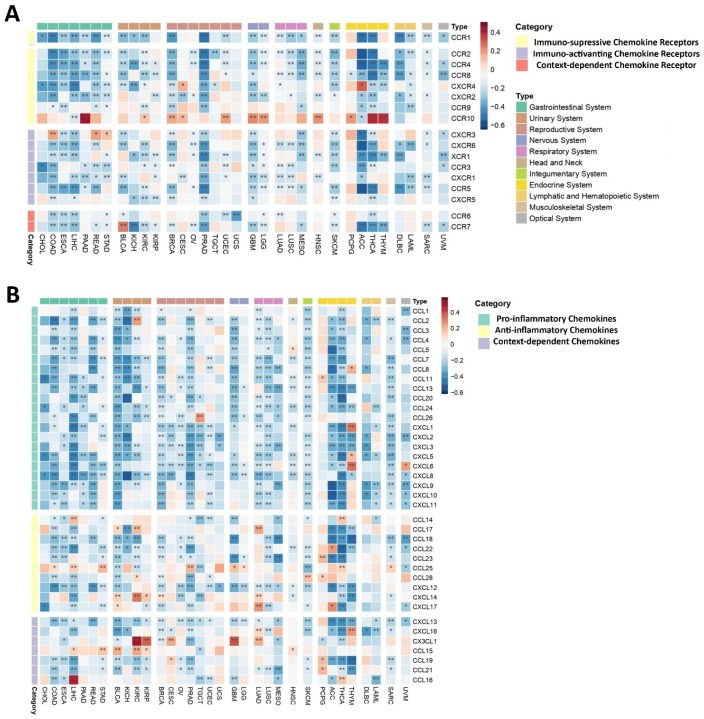
Heatmaps displaying correlations between *PEBP1/STK11* co-expression and the transcript levels of chemokine-related genes. (**A**) Chemokines receptors and (**B**) chemokines. Color coding: red, positive correlation; blue, negative correlation. * *p* ≤ 0.05, ** *p* ≤ 0.01.

Given the bifunctional nature of both chemokines and their receptors within the TME, as they can exhibit both pro-inflammatory and anti-inflammatory effects, depending on the specific physiological context, our analysis revealed a predominantly negative correlation between the *PEBP1/STK11* co-expression pattern and the so-called immunosuppressive chemokine receptor genes across most of the analyzed cancer types (Figure 4A). However, exceptions were observed in certain cancers, including *CXCR4* in CESC and adrenocortical carcinoma (ACC), as well as *CCR10* in various malignancies, such as nervous system cancers, endocrine malignancies (excluding ACC), PAAD, KIRC, BRCA, CESC, UCEC, and HNSC, where these chemokine genes demonstrated a positive correlation with *PEBP1/STK11* co-expression. Similarly, immune-activating chemokine receptor genes tended to show a negative correlation with *PEBP1/STK11* co-expression across all cancer types examined. The notable exception of this trend was observed in COAD, READ, and STAD, where *CXCR3* displayed a positive correlation with *PEBP1/STK11* expression, highlighting the complex and context-dependent interplay between *PEBP1/STK11* and chemokine-mediated immune regulation in the tumor microenvironment.

Accordingly, pro-inflammatory chemokine genes exhibited a broad negative correlation with *PEBP1/STK11* co-expression across cancers of the gastrointestinal, reproductive, nervous, respiratory, and hematopoietic systems, suggesting that *PEBP1/STK11* a potential role for in dampening inflammatory signaling within these malignancies (Figure 4B). Notably, endocrine cancers displayed the highest frequency of positive correlations with pro-inflammatory chemokine genes, particularly with *CCL8*, *CCL11*, *CXCL1*, *CXCL5*, and *CXCL6*, thus indicating a distinct regulatory pattern in this cancer subtype.

In contrast, the correlation patterns between genes encoding anti-inflammatory chemokines and *PEBP1/STK11* co-expression were more variable. *CCL18*, *CCL22*, *CCL23*, and *CXCL12* consistently showed a negative correlation across most cancer types, while other anti-inflammatory chemokine genes displayed a more complex, context-dependent relationship, with both positive and negative correlations depending on the specific malignancy (Figure 4B). Notably, *CCL25*, *CCL28*, *CXCL14*, and *CXCL17* were most frequently associated with positive correlations. The cancers exhibiting the strongest positive correlations between anti-inflammatory chemokine genes and *PEBP1/STK11* co-expression were found in the urinary, respiratory, and endocrine systems. Additionally, *CX3CL1*, a gene that encodes a context-dependent chemokine, showed a strong positive correlation with the co-expression of *PEBP1/STK11* in KIRC, KIRP, CESC, LUAD, and GBM, while *CCL16* exhibited a positive correlation only in LIHC and THCA. In contrast, *CXCL13* was negatively associated with *PEBP1/STK11* co-expression in almost all cancer types.

#### 2.3.2. Metabolic Niche

**Figure 5:**
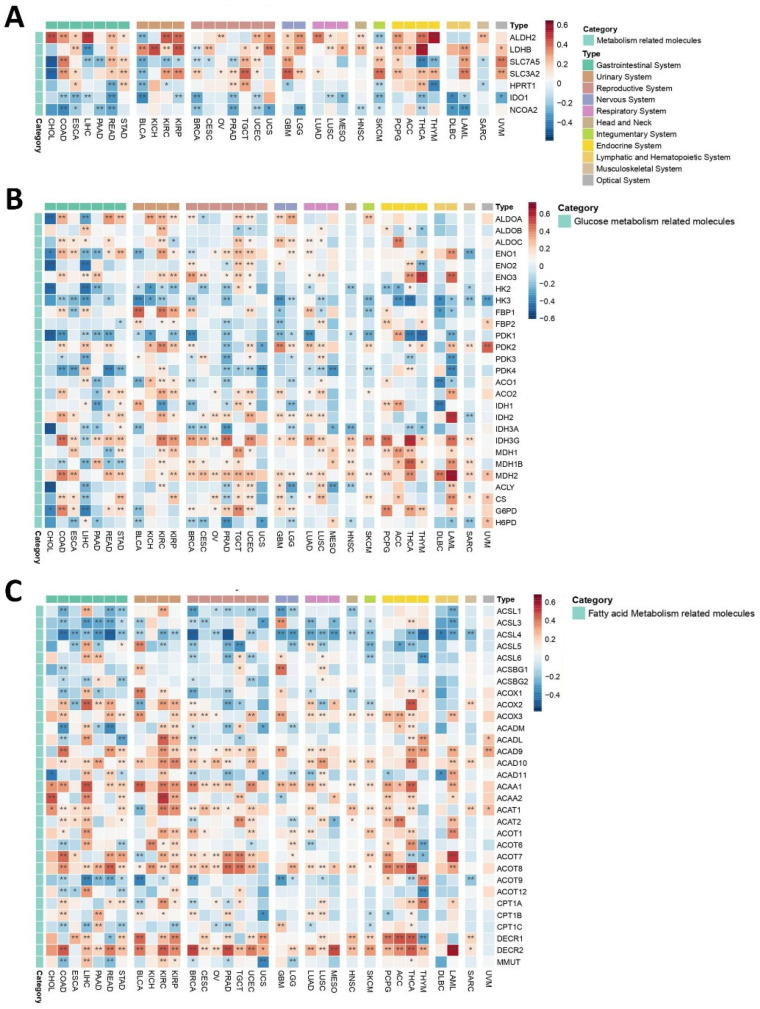
Heatmaps illustrating the effect of *PEBP1/STK11* co-expression on the transcriptional remodeling of the metabolic microenvironment. (**A**) Expression of general metabolism-related genes, (**B**) expression of genes involved in glucose metabolism, and (**C**) expression of genes associated with fatty acid metabolism. Color coding: red, positive correlation; blue, negative correlation. * *p* ≤ 0.05, ** *p* ≤ 0.01.

The metabolic sub-microenvironment (MM) of a TME is one of its major components, serving as a critical energy source that plays a pivotal role in shaping tumor growth, progression, and response to therapy. Understanding how *PEBP1/STK11* co-expression may interfere with the function and properties of tumor MM is essential for gaining deeper insights into tumor metabolism and its potential therapeutic implications. Among metabolism-related genes, *IDO1* and *NCOA2*, responsible for aggressive tumor characteristics, exhibited consistently negative correlations with *PEBP1/STK11* expression across most cancer types, thus suggesting that these genes may be involved in inhibitory metabolic pathways that counteract tumor progression in the presence of *PEBP1/STK11* co-expression (Figure 5A). In contrast, genes such as *ALDH2*, *LDHB*, *SLC7A5*, *SLC3A2*, and *HPRT1* displayed more complex patterns, with both positive and negative correlations observed in relation to *PEBP1/STK11* co-expression, indicating that these genes might be part of more dynamic metabolic processes and reprogramming that fluctuate depending on the tumor type and the *PEBP1/STK11* expression levels. Notably, *ALDH2*, a key metabolic reprograming gene, and *LDHB*, which plays a potential role in lactate metabolism, showed the strongest positive associations with *PEBP1/STK11* co-expression in LIHC, THYM, and KICH and THCA, respectively.

##### Glucose Metabolism

Glucose metabolism-related molecules exhibited a complex and heterogenous pattern of correlations with *PEBP1/STK11* co-expression, with both positive and negative associations observed across various cancer types (Figure 5B). A strong negative correlation was observed among genes associated with metabolic reprogramming in cancer cells such as *HK2*, *HK3*, and *PDK1*, which are involved in glycolysis and the co-expression of the aforementioned genes. More specifically, *HK3* displayed predominantly negative correlations across most cancer types, suggesting a widespread involvement of these enzymes in metabolic pathways influenced by *PEBP1/STK11* levels. In contrast, *ENO3*, *IDH2*, *IDH3G*, and *MDH2*, genes that encode proteins that generally represses the proliferative, migratory, and invasive capacities of cancer cells, displayed the strongest positive correlations across nearly all cancers.

Notably, CHOL and UCS cancers exclusively exhibited negative associations with all glucose metabolism-related genes that significantly correlated with *PEBP1/STK11* co-expression, highlighting a distinct metabolic phenotype in these cancer types. On the other hand, OV demonstrated an entirely opposite trend, with strong positive correlations between *PEBP1/STK11* co-expression and the majority of glucose metabolism genes, pointing to a unique metabolic signature in this cancer type.

##### Fatty Acid Metabolism

A similar complex and cancer type dependent pattern emerged when analyzing fatty acid metabolism-related genes, which made clear categorization challenging. Among these, *PEBP1/STK11* co-expression strongly negatively correlates in all cancer types with enzymes that increase the biosynthesis of fatty acids such as members of the ACSL family (*ACSL1*, *ACSL3*, *ACSL4*, *ACSL5*, and *ACSL6*). Particularly, *ACSL4* exhibited the most predominant negative correlation with *PEBP1/STK11* co-expression across multiple cancer types, suggesting an inhibitory role in fatty acid metabolism (Figure 5C). Such negative correlation was also observed with genes that encode enzymes that are responsible for fatty acid oxidation like CPT1 family members (*CPT1A*, *CPT1B*, and *CPT1C*). Similarly, a negative association was also established for the *MMUT* gene that encodes for methylmalonyl-CoA mutase in mitochondria, which plays a role in the regulation of energy-producing centers [69]. In contrast a strong positive association was revealed for *ACAA1* and *ACAA2*, which encode for enzymes that catalyze fatty acid oxidation, and for DECR family members (*DCR1* and *DCR2*), which encode for reductases that participate in FA metabolism. Additionally, *ACOX3*, *ACAD9*, *ACAD10*, *ACOT1*, *ACOT8,* and *DECR2* displayed positive associations with *PEBP1/STK11* co-expression across various cancer types, highlighting a potential involvement in enhancing fatty acid metabolism in the context of *PEBP1/STK11* expression.

These findings underscore the importance of examining the metabolic sub-microenvironment in greater detail, as it holds critical implications for understanding how *PEBP1/STK11* co-expression impacts tumor biology and could offer new avenues for targeted therapeutic interventions.

#### 2.3.3. Mechanical Niche

**Figure 6:**
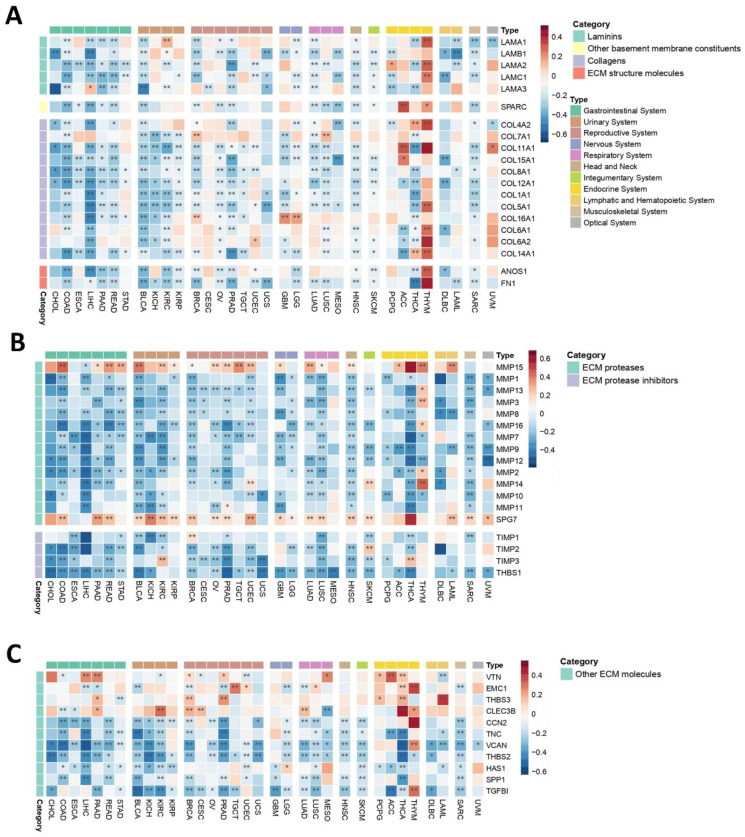
Heatmaps demonstrating how *PEBP1/STK11* co-expression modulates the transcriptional landscape of the mechanical microenvironment across 33 cancer types. (**A**) Genes encoding basement membrane components, (**B**) genes involved in ECM proteolysis, and (**C**) genes associated with ECM structure and functions. Color coding: red, positive correlation; blue, negative correlation. * *p* ≤ 0.05, ** *p* ≤ 0.01.

The mechanical microenvironment (MeM) is a critical TME niche as it affects intracellular signaling events, carcinogenesis, cancer progression, and tumor response to therapy [70]. Laminins, key components of the extracellular matrix (ECM), exhibited predominantly negative correlations with *PEBP1/STK11* co-expression across most cancer types (Figure 6A). Notable exceptions were observed for *LAMA1* in KIRP, *LAMA2* in PCPG, *LAMA3* in LIHC, and several laminins in THYM. Similarly, collagens and other ECM structural components displayed largely negative correlations with *PEBP1/STK11* co-expression, with the most frequent exceptions found in endocrine cancers, particularly in THYM.

To further explore the impact of *PEBP1/STK11* co-expression on MeM properties, we analyzed ECM proteases and ECM protease inhibitors (Figure 6B). ECM proteases generally showed a widespread negative correlation with *PEBP1/STK11* co-expression across all cancer types, with the exception of *MMP15* and *SPG7*, which displayed positive correlations in most of the cancers analyzed. Similarly, most ECM protease inhibitors exhibited negative correlations with *PEBP1/STK11* co-expression, with only a few exceptions, including *TIMP2* in SKCM and *TIMP3* in KIRC and THCA, where positive associations were observed.

Further analysis of additional ECM molecules, including *VTN*, *EMC1*, *THBS3*, and *CLEC3B* revealed a positive correlation trend with *PEBP1/STK11* co-expression in endocrine cancers, while correlations in other cancer types varied, suggesting a context-dependent relationship (Figure 6C). Furthermore, *CCN2*, *TNC*, *VCAN*, *THBS2*, *HAS1*, *SPP1*, and *TGFB1* gene expression was also analyzed, showing a predominantly negative trend in accordance with *PEBP1/STK11* co-expression, with the exceptions of *CCN2*, *VCAN*, and *TGFB1* in THYM and *HAS1* in LGG.

#### 2.3.4. Hypoxic Niche

**Figure 7:**
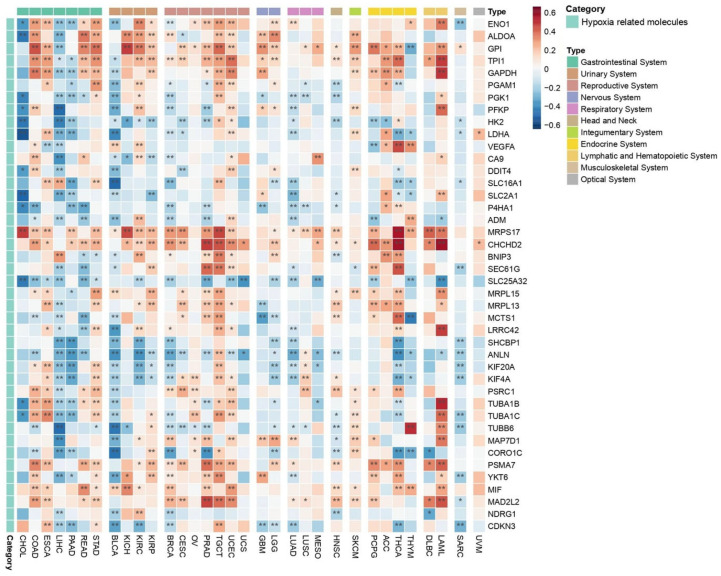
Heatmap depicting the effects of *PEBP1/STK11* co-expression on the transcriptional remodeling of the hypoxic microenvironment. Color coding: red, positive correlation; blue, negative correlation. * *p* ≤ 0.05, ** *p* ≤ 0.01.

A key aspect to consider within the TME is the hypoxic niche, which plays a critical role in tumor progression, metastasis, and therapeutic resistance. In this context, a total of 42 hypoxia-related genes (hypoxia-buffa signature) were analyzed, revealing complex correlation patterns across different cancer types, with variations influenced by context-dependent factors (Figure 7). Each cancer type displayed a mix of positive and negative correlations among the analyzed molecules, complicating the ability to draw broad, uniform conclusions. Nevertheless, in several cancers, hypoxia-related molecules generally showed a negative correlation with the co-expression of *PEBP1/STK11,* including CHOL, LIHC, PAAD, BLCA, BRCA, UCS, LUAD, and SARC.

Conversely, in other cancer types such as COAD, ESCA, READ, STAD, KICH, KIRC, KIRP, LUSC, MESO, HNSC, SKCM, and UVM, as well as cancers of the reproductive (excluding BRCA and UCS), nervous, endocrine, and hematopoietic systems, hypoxia-related molecules predominantly exhibited positive correlations with *PEBP1/STK11* co-expression. Among the hypoxia-related molecules, *MRPS17*, *CDCHD2*, *PSMA7*, and *MIF* consistently demonstrated a positive correlation with *PEBP1/STK11* co-expression across all cancer types in which a significant association was observed. In contrast, *SHCBP1* gene that exhibits a dysregulated expression across a broad spectrum of malignancies and contributes to multiple hallmarks of cancer, including enhanced cellular proliferation, resistance to apoptosis, and increased invasive and metastatic potential, which displayed a predominantly negative correlation in most cancer types. This indication suggests the potential role of *PEBP1/STK11* co-expression in tumor suppression or altered metabolic regulation in hypoxic conditions.

Genes like *PGK1*, *PFKP*, *HK2*, and *LDHA*, that are involved in metabolic reprogramming of cancer cells by enhancing glycolysis have a negative correlation with *PEBP1/STK11* co-expression. Accordingly, *ANLN*, *KIF20A*, and *KIF4A*, involved in the regulation of cytoskeletal dynamics for cell migration and division, seem to display a negative correlation with the co-expression of the metastasis inhibitors as well. Similarly, a negative correlation was also observed with LSC transporters genes (*SLC16A1*, *SLC2A1*, and *SLC25A32*), which are primarily involved in metabolite transport across cell membranes. However, a positive correlation was identified in molecules that promote tumor progression like *VEGFA*, *ALDOA*, and *GPI*.

These findings emphasize the heterogenous nature of the TME and the differential regulatory roles of hypoxia-related molecules across various cancer types, as well as the context-specific interactions between hypoxia, *PEBP1*, and *STK11* that may provide insights into tumor biology and potential therapeutic strategies, emphasizing in reprogramming of hypoxic niches.

### 2.4. PEBP1/STK11 Co-Expression Modulates TME Properties and Therapeutic Responses

#### 2.4.1. EMT

**Figure 8:**
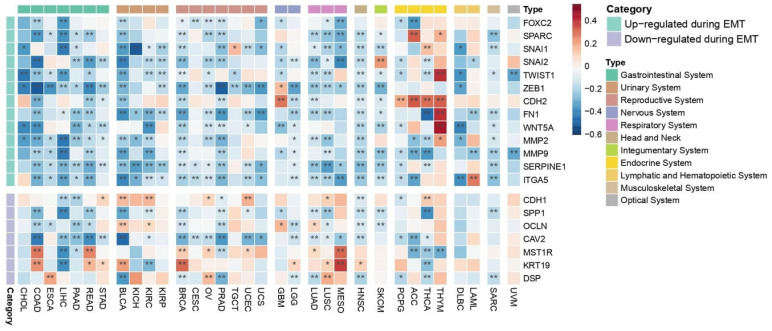
Heatmap illustrating the impact of *PEBP1/STK11* co-expression of EMT-associated genes across cancer types. Color coding: red, positive correlation; blue, negative correlation. * *p* ≤ 0.05, ** *p* ≤ 0.01.

Oncogenic EMT, known as a precursor event to cancer cell metastasis, is a dynamic process which is regulated by both molecular alterations in cancer cells towards and the mechanical properties of the TME, which facilitate local cancer cell invasion [71]. Our analysis revealed that genes that encode molecules that are commonly up-regulated during EMT such as *FOXC2*, *SNAI1*, *SNAI2*, *TWIST1*, *ZEB1*, *FN1*, *WNT5A*, *MMP2*, *MMP9*, *SERPINE1*, and *ITGA5* generally exhibit a negative correlation with the co-expression of *PEBP1/STK11* across most cancer types, with a few exceptions (Figure 8). Endocrine cancers, however, display a more complex pattern, with both positive and negative correlations observed between the up-regulated EMT-associated molecules and the co-expression of the studied genes. Conversely, genes that encode molecules that are down-regulated during EMT such as *CDH1*, *OCLN*, and *DSP*, tend to show stronger positive correlations than the up-regulated ones. A mixed phenotypic pattern emerges, characterized by both positive and negative correlations, with the most statistically significant positive correlations found for *MST1R* and *KRT19* in COAD, READ, BRCA, and MESO. Those genes act as regulators of cellular signaling networks that drive tumor development and support cancer cell survival [72,73]. Notably, *SPP1,* which facilitates cancer cell chemoresistance [74] and *CAV2*, which facilitates cellular invasion and migration while suppressing the expression of tumor-suppressor genes [75], exhibited a consistent negative correlation with the co-expression of *PEBP1/STK11* in most cancer types where a statistically significant correlation was observed, suggesting a broader, potentially universal relationship. Contrastingly, the gene encoding e-cadherin (*CDH1*), a well-established tumor suppressor protein, displayed a positive correlation with *PEBP1/STK11* co-expression in STAD, BLCA, KIRC, OV, UCEC, LUSC, and THCA.

#### 2.4.2. Predictive Response to Chemotherapy

**Figure 9:**
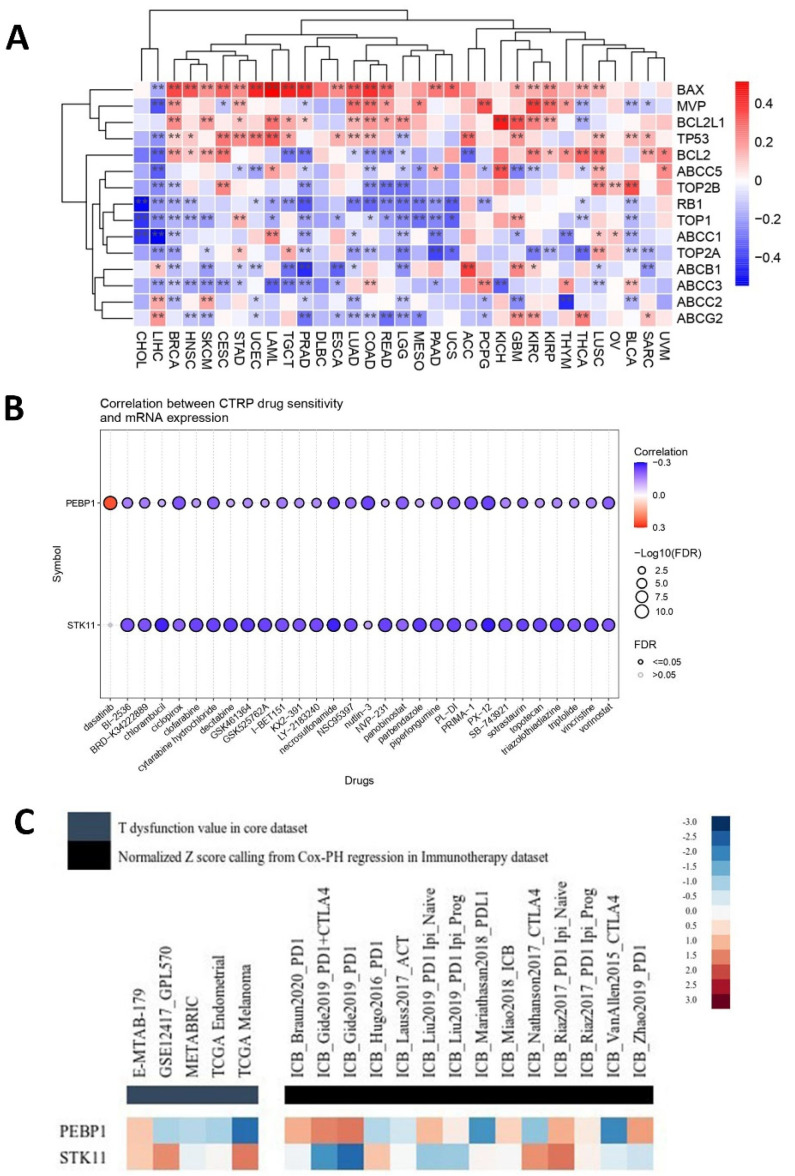
Analysis of the *PEBP1/STK11* expression signature in relation to therapeutic response. (**A**) Heatmap depicting associations with genes linked to drug resistance, (**B**) predictive value of *PEBP1* and *STK11* expression levels for drug sensitivity across various cancer types, and (**C**) regulator prioritization clustering heatmap showing the associations between *PEBP1/STK11* transcript levels and T-cell dysfunction, as well as responses to ICBs. Color coding: red, positive correlation; blue, negative correlation. * *p* ≤ 0.05, ** *p* ≤ 0.01.

The TME confers to cancer chemoresistance, with the individual niches within the TME contributing in distinct ways. Given the interplay between *PEBP1/STK11* and TME niches, as we demonstrated above, it is essential to further investigate how drug resistance may be influenced by the co-expression of these genes. Several genes associated with drug resistance, particularly those involved in drug efflux, such as members of the ATP binding cassette (ABC) transporter family, including *ABCC1*, *ABBC2*, *ABBC3*, *ABCC5*, *ABCB1*, and *ABCG2*, displayed a general trend of significant negative associations with *PEBP1/STK11* co-expression across the majority of cancer types, with a few exceptions observed mainly in LIHC and GBM (Figure 9A). Similarly, *MVP* and genes encoding for Type I and Type II topoisomerases, such as *TOP1, TOP2A*, and *TOP2B*—known to contribute to cancer chemoresistance—showed the same negative association pattern. Other genes such as the pro-apoptotic gene *BAX*, as well as the tumor suppressor *TP53*, showed strong statistically significant positive correlations in most cancer types, while the antiapoptotic *BCL2L1* and *BCL2* genes presented a more intricate pattern, exhibiting both positive and negative correlations with *PEBP1/STK11* co-expression on a cancer type dependent manner. On the other hand, the tumor suppressors *TP53* and *RB1* exhibited contrasting correlation patterns with *PEBP1/STK11* co-expression, with *TP53* generally showing a positive correlation, while *RB1* was the only gene that consistently exhibited a negative correlation across the majority of cancer types analyzed.

Furthermore, a predictive drug sensitivity analysis was conducted, to evaluate the impact of *PRBP1* and *STK11* transcript levels on drug responsiveness. A predominantly negative correlation was identified between the mRNA expression of both genes and sensitivity to a wide range of anti-cancer agents, including chemotherapeutics (e.g., Chlorambucil), targeted therapies (Panobinostat), and experimental inhibitors (e.g., GSK461364) [76], across a pan-cancer dataset (Figure 9B). This negative correlation indicates that the higher expression levels of *PEBP1* and *STK11* are associated with increased drug sensitivity across various cancer types. Notably, while the individual contribution of each gene may vary, the overall trend remains consistent across all tested agents, reinforcing a potential shared modulatory effect on therapeutic resistance

Overall, it appears that the co-expression of *PEBP1* and *STK11*, in conjunction with specific drug resistance-related molecules within the TME and particular therapeutic agents, may critically influence therapeutic responses.

#### 2.4.3. Predictive Response to Immunotherapy

*PEBP1* and *STK11* transcript levels were associated with immunosuppressive properties and responses to immune checkpoint blockade (ICB) therapies, such as anti-PD1 and anti-CTLA4, particularly in melanoma, kidney, and lung cancers. The regular prioritization analysis using the TIDE framework highlights distinct immunogenomic profiles for *PEBP1* and *STK11* across multiple datasets (Figure 9C). Both genes show variable normalized Z-scores derived from Cox proportional hazards regression and T-cell dysfunction scoring. Specifically, *PEBP1* displayed moderate to strong inverse associations with T-cell dysfunction, while showing elevated scores in datasets associated with specific immunotherapy cohorts using different ICBs. In contrast, *STK11* presented a more heterogeneous profile, with mixed associations across datasets showing potential involvement in responses to ICBs but with less consistent prioritization compared to *PEBP1*. Notably, both genes seem to be linked to M2 macrophage and CAF-associated expression profiles, suggesting a potential role in immune evasion and tumor microenvironment remodeling.

These results indicate that *PEBP1* may be a stronger candidate for immunotherapy effectiveness prediction, while *STK11* may contribute to immune modulation in a more context-dependent manner.

#### 2.4.4. Pathway Analysis

**Figure 10:**
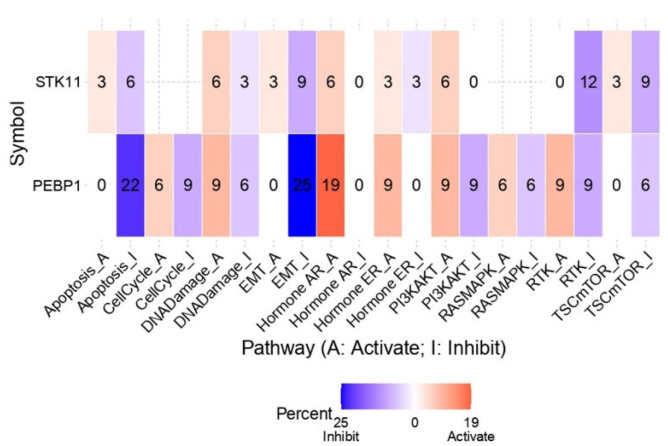
Impact of *PEBP1* and *STK11* expression on the activity of 10 cancer-related pathways. The heatmap and linked numbers display the percentage of tumor types in which *PEBP1* and *STK11* mRNA expression is significantly associated with either activation or suppression of each pathway. Color coding: red, pathway activation; blue, pathway inhibition.

An integrated analysis was further conducted to assess the regulatory impact of the *PEBP1/STK11* co-expression signature on ten major cancer-associated pathways across diverse cancer types. The results indicate that *PEBP1* mRNA levels are strongly associated with suppression of the apoptosis inhibition (22%) and EMT (25%) pathways across the pan-cancer landscape (Figure 10). This suppressive effect appears to be further enhanced by *STK11* expression, which contributes an additional 6% and 9% reduction, respectively. Interestingly, both *PEBP1* and *STK11* were also found to induce the androgen receptor (AR) activation pathway by 19% and 6%, respectively.

Regarding oncogenic signaling pathways with key roles in regulating cancer cell growth, proliferation, survival, and metabolism, including PI3K/AKT, RAS/MAPK, RTK, and TSC/mTOR, a consistent pattern emerged. *PEBP1* expression was distinctly linked to the inhibition of all four pathways, with additionally inhibitory contributions from *STK11* expression, especially in the RTK and TSC/mTOR pathways. Although we also observed a minor activating impact of *PEBP1/STK11* on the aforementioned pathways, the inhibitory effect was substantially stronger than the activating effect, suggesting that *PEBP1/STK11* co-expression mainly influence negatively the molecular context examined.

## 3. Discussion

This study aimed to explore the co-expression of RKIP and LKB1 transcripts across human cancers included in the TCGA database (accessed on 17 May 2025), hypothesizing that their joint expression may define a common molecular signature that shapes key TME niches. Rather than drawing definitive conclusions, we concentrated on combining existing biological knowledge with publicly available multi-omics data to produce testable hypotheses regarding the potential modulation of TME characteristics and the impact of these genes on clinically relevant outcomes. The computational framework described here is meant to support hypothesis-driven investigation of crucial biomarkers and guide future experimental validation.

Both RKIP and LKB1 are widely recognized as tumor-suppressing proteins, with established, yet largely non-redundant functions in tumor biology [11,77]. RKIP primarily modulates signaling pathways such as RAF/MEK/ERK, NF-κB, and STAT3, affecting inflammatory responses, EMT, and apoptosis resistance. In contrast, LKB1 regulates metabolic homeostasis and energy stress via AMPK, while also influencing immune evasion by preventing the epigenetic silencing of STING and modulating cytokine production. Despite their protective functions, both proteins are frequently downregulated or even lost in a wide range of malignancies, often correlating with more aggressive tumor characteristics, poor prognosis, and therapeutic resistance. Recent studies present evidence suggesting that RKIP and LKB1 independently influence the TME through distinct, yet complementary, mechanisms. Together, their co-expression may represent a convergence of metabolic regulation and immunomodulatory signaling that synergistically suppresses tumor progression and reprograms the TME toward an anti-tumor phenotype.

RKIP’s role in remodeling the immune TME has been increasingly recognized, particularly through its regulation of immune cell infiltration—most notably macrophages—and its control over the secretion of pro-metastatic factors [78,79]. Similarly, loss-of-function mutations in LKB1 have been associated with reduced PD-L1 expression in tumor cells, as well as decreased infiltration of cytotoxic CD8^+^ T cells [80]. In line with these findings, our current analysis provides further evidence that the combined expression of *PEBP1* and *STK11* may favor an immune-permissive TME at a cancer type specific context; however, these findings merit further attention and deeper examination. More specifically, their co-expression was negatively correlated with M2 macrophages, which are generally associated with immunosuppression, and positively correlated with CD8^+^ T and NK cell infiltration across most cancer types.

RKIP has been further reported to suppress the expression of pro-inflammatory cytokines and chemokines by inhibiting NF-κB signaling, thereby limiting chronic inflammation, tumor invasion, and metastatic dissemination [27,78,81]. Similarly, LKB1 has been implicated in the regulation of chemokine signaling pathways, contributing to immune regulation within the TME [82]. In the present study, co-expression of *PEBP1/STK11* was predominantly negatively correlated with several pro-inflammatory chemokines and immunosuppressive chemokine receptors, while showing positive correlations with anti-inflammatory chemokines in a cancer type dependent manner. This expression pattern suggests a synergistic role for these tumor suppressors in both mitigating chronic inflammation and enhancing immune surveillance against cancer cells, providing clear indications of potential clinical significance that warrant experimental investigation. In the context of immune inhibitory signals, molecules such as PD-L1 and PD-L2 that are known to contribute to an immunosuppressive TME, have been previously reported to be inversely associated with RKIP and LKB1 expressions [83,84,85]. Consistent with these findings, our analysis revealed uniformly negative associations between *PEBP1/STK11* co-expression and the expression of genes encoding immune checkpoint molecules across all cancer types examined, suggesting that their co-expression may contribute to the development of a more immunocompetent and less suppressive TME. Furthermore, in line with prior studies demonstrating that both RKIP and LKB1 individually suppress TNF-α expression [30,86,87,88], our data show a negative correlation between TNF ligand expression and *PEBP1/STK11* co-expression. These preliminary findings further underscore the potential role of these tumor suppressors in limiting constitutive, inflammation-driven tumor progression that can be leveraged to formulate biologically meaningful predictions.

In addition to the transcriptional remodeling of the immune niche within the TME, *PEBP1/STK11* co-expression was also associated with significant alterations in metabolic and hypoxic TME subtypes. Specifically, co-expression was negatively correlated with transcripts involved in metabolic reprogramming, particularly those related to fatty acid biosynthesis and oxidation. This pattern provides early-stage evidence of a potential reduction in energy availability within cancer cells, possibly contributing to mitochondria destruction, metabolic stress, and decreased cell survival, requiring experimental validation. However, despite the complexity and inconsistencies observed in the context-dependent associations across different cancer types—especially regarding particular genes with reported role in fatty acid metabolism—a notable exception was the positive association between *PEBP1/STK11* co-expression and *ACAA1* levels. This observation aligns with recent studies identifying ACAA1 as a favorable prognostic marker, linked to apoptosis induction and enhanced T-cell infiltration [89], thus further linking metabolism to immune regulation within the TME and highlighting the role of *PEBP1/STK11* co-expression on this interplay.

*PEBP1/STK11* co-expression was further shown to modify the mechanical and structural components of the TME across human cancers. A particularly strong and uniform negative correlation was evidenced between the co-expression of these genes and the transcription of several major matrix metalloproteinase (MMPs) and *PEBP1/STK11* levels, indicating a suppressive effect on local tumor invasion. This observation is in line with previous reports showing that RKIP and LKB1 can individually downregulate MMP’s expression levels through multiple signaling pathways (e.g.: Raf/MEK/ERK, NF-κB) [27,90]. Moreover, our epithelial-to-mesenchymal transition (EMT) analysis revealed a consistent inverse association between *PEBP1/STK11* co-expression and EMT marker genes, including *SNAI1, SNAI2*, *FOXC2*, *ZEB1*, and *FN1,* across most cancer types examined, thus underscoring a uniform anti-EMT signature. Additionally, genes like *SPP1* and *CAV2* that are involved in drug resistance and cellular invasion and migration, respectively, were also found to have a negative association with the co-expression of the examined genes. While RKIP is recognized as an EMT inhibitor [11,12,17,91,92], the precise mechanisms of its regulatory role remain unclear. Likewise, LKB1 inactivation has been implicated in promoting EMT [12,93]. Overall, our findings provide new evidence supporting the hypothesis that high *PEBP1/STK11* co-expression may be involved in suppressing EMT-related features of cancer cells within the TME, which opens new avenues for hypothesis-driven research for the remodeling of the TME.

In parallel, LKB1 silencing has been reported to enhance cancer cell adhesion to ECM components, such as laminin, collagen IV, and fibronectin [94]. On the other hand, RKIP has been shown to promote the expression of epithelial markers and adhesion molecules, such as E-cadherin, laminin, and EPCAM [12,91,95]. Additionally, it has been shown that locostatin-mediated RKIP inhibition leads to a decrease in ECM components [96]. In our analysis, *PEBP1/STK11* co-expression was negatively correlated with multiple genes encoding ECM components, suggesting a shift towards a less rigid and more permeable TME that may facilitate easier immune cell infiltration. Collectively, our data support a shared expression signature between RKIP and LKB1 in restructuring the mechanical properties of the TME in a way that limits local invasion of cancer cells while promoting immune cell accessibility. The presented evidence aligns with current research trajectories and strengthens the rationale for clinical exploration.

Beyond its established role in ΕΜΤ and metastasis suppression, RKIP has also been reported to sensitize tumor cells to conventional chemotherapy, radiotherapy, and endogenous immune-mediated cytotoxicity [11]. Similarly, loss of LKB1 function has been associated with aggressive tumor behavior and resistance to multiple therapies, including chemotherapy, targeted treatments, and immune checkpoint inhibitors [97]. In line with these observations, our TME-focused analysis further implicates *PEBP1/STK11* co-expression in modulating therapy resistance. Specifically, these findings indicate that high levels of co-expression were associated with lower expression of genes enhancing drug resistance (e.g., ABC transporters and topoisomerases of Type I/II), thus suggesting a potential prognostic value in predicting a favorable response to conventional chemotherapies. Collectively, these early-stage, integrative bioinformatic insights offer a strong foundation for generating testable hypotheses in the context of cancer therapy.

Consistently, drug sensitivity prediction analysis revealed a robust, uniform association between high expression of both genes and increased sensitivity to all major classes of chemotherapeutic agents, across all tumor types examined. This consistent pattern suggests that the coordinated expression of these tumor suppressors enhances tumor efficacy. Mechanistically, these findings are supported by previous reports indicating that high RKIP expression dampens pro-survival signaling pathways, such as RAF/MEK/ERK and NF-κB, while it disrupts oncogenic feedback loops, thus making cancer cells more prone to drug-induced apoptosis [98,99]. Likewise, high *STK11* expression contributes to therapeutic vulnerability by activating AMPK and inhibiting mTOR signaling, which together enhance cellular stress responses, attenuate survival pathways, and increase apoptosis [84,100]. These observations were further corroborated by pathway enrichment analysis, which demonstrated that concurrent high expression of both genes was associated with suppression of apoptosis inhibition, EMT, and several key oncogenic signaling pathways such as PI3K/AKT, RAS/MAPK, RTK, and TSC/mTOR. Altogether, these findings point to a synergistic role of RKIP and LKB1 in sensitizing tumors to chemotherapy by reshaping the TME towards a more therapeutically responsive state. In contrast the synergistic effect of both genes in predicting immune activation and responses to immunotherapy agents such as ICBs, it was more heterogenous and less conclusive across certain cancers. However, compared to *STK11*, the *PEBP1* expression signature appears to be a more reliable predictor of T-cell function and immunotherapy outcomes in the context of ICB treatment. Hence, these initial findings outline a promising direction that should be substantiated through rigorous experimental and clinical validation.

Despite these promising findings, several limitations of our study must be acknowledged. First, the bifunctional nature of chemokines and their receptors within the tumor microenvironment (TME) presents a significant interpretive challenge. Chemokines are highly context-dependent; a single molecule can exert either pro- or anti-inflammatory effects depending on tumor type, immune landscape, and temporal dynamics. Additionally, the promiscuity of chemokine receptors, where one receptor may bind multiple ligands, can lead to overlapping or even antagonistic functional outcomes, complicating the precise characterization of immune signaling within the TME.

A second major limitation is the exclusive reliance on transcriptomic data from TCGA, which, while rich and comprehensive, does not account for post-transcriptional regulation or post-translational modifications. As such, our conclusions should be interpreted as correlative and hypothesis-generating. In addition, while our findings strongly suggest functional links, they remain purely correlative, highlighting a clear need for in vitro and even in vivo validation using functional assays, protein-level measurements, and spatial profiling to confirm the biological relevance of the observed associations. In this context, several experimental approaches could be applied to selected patterns of interest. For example, as noted above, our in silico analysis revealed a robust pan-cancer negative correlation between the co-expression of RKIP and LKB1 and a range of immune inhibitory genes, including key immune checkpoint molecules (Figure 3). One would select human cancer cell lines (e.g., lung or pancreatic adenocarcinomas or melanomas) characterized by low to moderate endogenous expression of RKIP and LKB1. These lines would then undergo genetic manipulation: individual or combined gene overexpression (via CRISPRa) or knockdown/suppression (via CRISPRi). Subsequently, transcriptomic profiling—using RNA-seq or selected qPCR panels—would measure expression of those immune-inhibitory genes that displayed inverse associations in silico, alongside other relevant genes. These transcriptional changes would be validated at the protein level using CyTOF or classical flow cytometry to assess expression not only the tumor cells but also in relevant surface markers on immune cells (e.g., CD8^+^ T cells or dendritic cells) co-cultured with the modified cancer cells. In the co-culture system, we would also measure functional immune readouts, such as proliferation, cytokine release (e.g., IFN-γ, IL-2), and indicators of cytotoxicity: Granzyme B levels, LDH release, etc. Moreover, mechanistic experiments would probe phospho-signaling events and downstream pathways identified by our in silico analysis as mediators of RKIP and LKB1’s regulatory effects on immunomodulatory gene expression. These mechanistic insights underscore the value of moving beyond correlative pan-cancer associations to targeted experimental validation of RKIP and LKB1’s roles in regulation of immunosuppressive molecules and signaling pathways.

Third, the *PEBP1/STK11* co-expression signature used in this analysis captures the combined effect of the two genes but does not disentangle their individual contributions or the mechanistic basis of their interplay. It remains unclear whether their apparent synergy reflects convergent regulation of shared pathways or distinct, complementary functions acting on different components of the TME. Furthermore, while we report associations with survival and therapy response, these are based on retrospective analyses, and no causal inference can be made. Finally, although this study draws from a large pan-cancer dataset, the heterogeneity of the TCGA samples, including uneven sample sizes across tumor types, potential batch effects, and missing clinical annotations, may influence the statistical power and generalizability of certain findings. Moreover, the use of correlation-based frameworks such as GSVA assumes primarily linear relationships and may not capture more complex, nonlinear regulatory dynamics. In light of these limitations, the primary value of our study lies in its ability to integrate prior biological knowledge with large-scale multi-omics data to generate testable hypotheses.

Concluding, the co-expression of *PEBP1/STK11* appears to play a central role in remodeling the TME by regulating immune cell infiltration, ECM structure, metabolic reprogramming, and therapy response. Their synergistic expression promotes an immune- permissive, anti-inflammatory, and less invasive TME, potentially improving prognosis and therapy outcomes. These findings highlight *PEBP1/STK11* co-expression as a promising therapeutic axis and prognostic marker. Importantly, this work illustrates how prior knowledge and curated datasets can be leveraged to formulate biologically meaningful predictions. Hence, further experimental validation and mechanistic studies are required to clarify their interplay and assess their translational potential in clinical oncology.

## 4. Materials and Methods

### 4.1. Protein Functional Analysis by the Multiple UniReD Tool

To identify potential functional relationships between RKIP and LKB1 proteins and proteins with an established role in drug resistance, hypoxia, metabolic, and mechanical tumor microenvironments, we employed the text-mining tool multiple UniReD [101]. Multiple UniReD utilizes the published biomedical literature to associate proteins of interest (query list) with a list of reference proteins known to be involved in a specific condition or disease (reference list) [102]. Each protein in the query list is assigned a score indicating its level of association with the reference list proteins. A maximum score of 1 is assigned when both query and reference proteins are found within the same UniReD cluster, indicating a strong functional connection. If a paralogue of the query protein has been found in the same cluster with a reference protein or an association was found between orthologues of the query and reference proteins, a score of 0.5 is given. A score of 0 indicates no detectable relationship in the literature. The higher the total score the higher the functional association of the proteins of interest to the reference list proteins.

### 4.2. Data Acquisition and Preprocessing

To study the effects of *PEBP1* and *STK11* co-expression and their role in shaping the TME in a pan-cancer setting we leveraged the TCGAplot R package (v8.0.0) to assemble and preprocess pan-cancer transcriptomics data from The Cancer Genome Atlas Project (TCGA). As stated in the original paper’s gene expression profiles (transcripts per million, TPM) for 33 TCGA tumors and normal tissues were downloaded from the GDC portal using the TCGAbiolinks R package (v2.28.4). Duplicate samples were removed and genes with no measurable expression (TPM = 0 in all samples) were filtered out, yielding an expression matrix restricted to protein-coding genes. Expression values were then log-transformed (reported as log_2_TPM + 1) and annotated with the corresponding cancer type and sample group (tumor or normal). All datasets were combined with sample meta-information (including clinical data and cancer type labels) for each TCGA case to enable comprehensive integrative pan-cancer analyses.

### 4.3. Exploring PEBP1 and STK11 Expression Signature

To assess the functional impact of *PEBP1* and *STK11* activity across multiple cancer types, we constructed a two-gene signature comprising these genes and performed Gene Set Variation Analysis (GSVA) using the transcriptomics data and core TCGAplot functions. GSVA scores were calculated using the GSVA function from the GSVA R package (v.2.0.5), with the *PEBP1/STK11* gene set supplied as a custom input. These enrichment scores reflected the relative activity of the two-gene module on a per-sample basis across all cancer types. Samples with high GSVA scores tend to have high expression of both genes, while those with low scores show low or discordant expression. Subsequently, we used the cor.test function (Pearson correlation) in R to examine associations between GSVA scores and various tumor microenvironment (TME) features, including immune cell infiltration, stromal content, and expression of immune regulatory genes. To characterize the tumor immune microenvironment, immune cell infiltration ratios were integrated from the TCGA Immune Landscape of Cancer study (downloaded via the GDC API). In addition, immune contexture scores (ESTIMATE, immune, and stromal scores) were calculated using the ESTIMATE algorithm (estimateR package v1.0.13) based on the log_2_TPM expression matrix. The resulting correlations were visualized and interpreted in a cancer-type specific context, revealing diverse interactions of the *PEBP1/STK11* module with immune, metabolic, mechanical, and hypoxic niches within the TME.

To facilitate reproducibility and support methodological transparency, we have provided a fully annotated R script as Supplementary Material (correlations.R). This script implements the core computational workflow used in our study, including GSVA score calculation, pan-cancer correlation analysis with immune/genomic/proteomic features, and heatmap visualization, enabling readers to reproduce our analyses or adapt them to their own gene sets and datasets.

### 4.4. Pathway Activity Analysis

We further explored the difference between ten cancer-related pathway activities (activation or inhibition) and *PEBP1/STK11* expression. To assess this pathway activity score (PAS), data of reverse phase protein array (RPPA) for 7876 samples across 32 cancer types was extracted from the TCPA portal (https://www.tcpaportal.org/tcpa, retrieved on 15 November 2023). Standard deviation was used to normalize these median-centered data of all samples. Subsequently, the PAS was computed as previously described [103].

The disparity in PAS between low and high expression groups was determined through the student’s t test. Subsequently, the *p* values were corrected for FDR, with the significance threshold set at 0.05, following the methodologies outlined previously [104,105]. When the sample displays increased gene expression, and simultaneously significantly elevated pathway activity (FDR ≤ 0.05), it is implied that the gene has potential stimulating activity on the pathway, and vice versa.

### 4.5. Correlation of PEBP1/STK11 Expression with Drug Sensitivity

To analyze the anti-cancer drug sensitivity, initially half maximal inhibitory concentration (IC50) data of 481 small molecules in 1001 cell lines was collected from the Genomics of Therapeutics Response Portal (CTRP) [106,107,108]. These data were then merged separately with their corresponding mRNA expression data for Pearson correlation analysis, offering the association between gene expression and IC50 of a particular drug. The *p*-values were also FDR-adjusted.

Univariate analysis was performed using the Pearson’s correlation between the *PEBP1* or *STK11* expression and Topotecan drug activity in different kidney, pancreatic, and lung cancer cell lines, using CellMinerCD [109].

Furthermore, the TIDE algorithm was further employed to identify the correlation between *PEBP1* and *STK11* mRNA expression and ICB therapy outcomes (https://tide.dfci.harvard.edu/ accessed on 21 May 2025) [110,111].

## Data Availability

All data available publicly at the GDC Data Portal (https://portal.gdc.cancer.gov/).

## Supplementary Materials

The following supporting information can be downloaded at: https://www.mdpi.com/article/10.3390/ijms26157145/s1.

## Abbreviations

LAML: acute myeloid leukemia; ACC, adrenocortical carcinoma; BLCA, bladder urothelial carcinoma; BRCA, breast ductal carcinoma; CESC, cervical carcinoma; CHOL, cholangiocarcinoma; COAD, colorectal adenocarcinoma; DLBC, diffuse large B-Cell lymphoma; ESCA, esophageal carcinoma; STAD, gastric adenocarcinoma; GBM, glioblastoma multiforme; HNSC, head and neck squamous cell carcinoma; LIHC, hepatocellular carcinoma; KICH, chromophobe renal cell carcinoma; KIRC, clear cell renal carcinoma; KIRP, papillary renal cell carcinoma; LGG, lower grade glioma; LUAD, lung adenocarcinoma; LUSC, lung squamous cell carcinoma; MESO, mesothelioma; OV, ovarian serous adenocarcinoma; PAAD, pancreatic ductal adenocarcinoma; PCPG, paraganglioma and pheochromocytoma; PRAD, prostate adenocarcinoma; READ, rectum adenocarcinoma; SARC, sarcoma; SKCM, skin cutaneous melanoma; TGCT, testicular germ cell cancer; THYM, thymoma; THCA, thyroid papillary carcinoma; UCS, uterine carcinosarcoma; UCEC, uterine corpus endometrioid carcinoma; and UVM, uveal melanoma.
