# Supplementary material for: Pan-Cancer Computational Analysis of RKIP (PEBP1) and LKB1 (STK11) Co-Expression Highlights Distinct Immunometabolic Dynamics and Therapeutic Responses Within the Tumor Microenvironment
Source: Int J Mol Sci. 2025 Jul 24;26(15):7145. doi: 10.3390/ijms26157145 (PMC12346703; doi:10.3390/ijms26157145)
Supplement: Supplementary file 1 [file ijms-26-07145-s001.zip › ijms-3741116-supplementary.pdf]

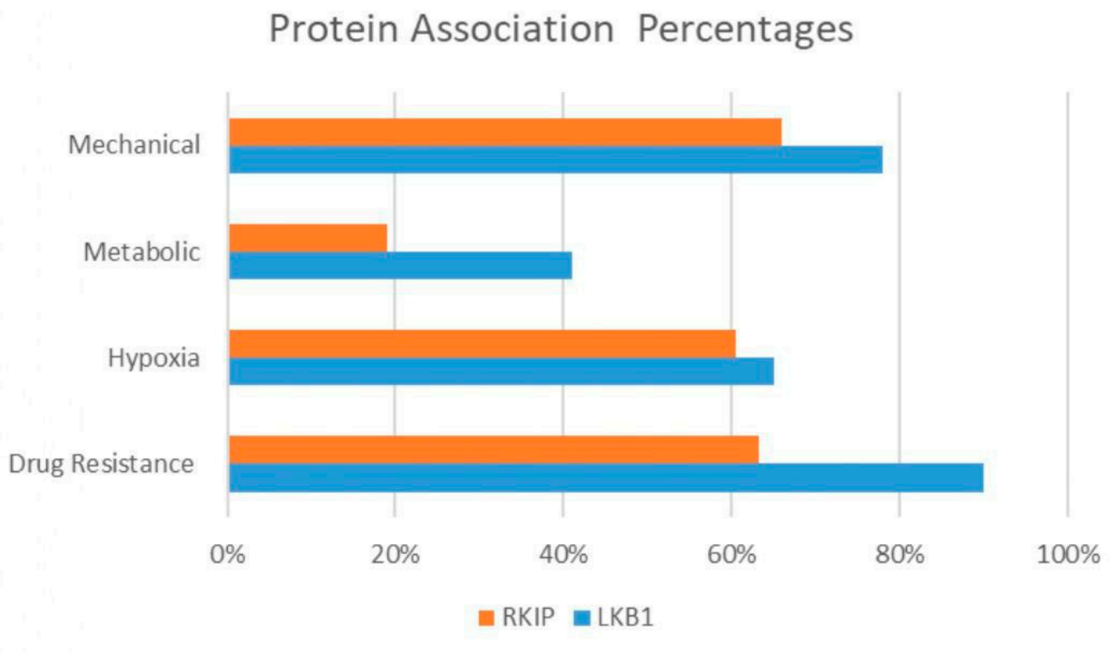

Supplementary Figure S1: Barchart depicting functional association score percentages between drug resistance, hypoxia, metabolic, and mechanical TME proteins with RKIP and LKB1, as determined by the text-mining tool Multi-UniReD.
